# Prevalence of Nocturnal Enuresis Among Children Dwelling in Rural Areas of Sindh

**DOI:** 10.7759/cureus.9590

**Published:** 2020-08-06

**Authors:** Muhammad Bilal, Abdul Haseeb, Alina Saeed, Aena Saeed, Tooba Sarwar, Sana Ahmed, Alina Ishaque, Maryam Raza

**Affiliations:** 1 Pediatrics, Dow Medical College, Dow University of Health Sciences, Karachi, PAK; 2 Internal Medicine, The Wright Center for Graduate Medical Education, Scranton, USA; 3 Internal Medicine, Ziauddin University, Karachi, PAK; 4 Internal Medicine, Dow Medical College, Dow University of Health Sciences, Karachi, PAK

**Keywords:** nocturnal enuresis, prevalence, risk factors, rural areas

## Abstract

Introduction

Nocturnal enuresis (NE) is usually a condition of childhood and refers to involuntary urinary incontinence during sleep. Due to its impact on a child’s mental and social health, it is important to determine the prevalence of this condition among a population. Therefore, the aim of our study is to evaluate its prevalence and associated risk factors among children living in rural areas of Sindh province, Pakistan.

Methods

Fifteen-hundred children aged between three and 12 years of age who lived in rural areas of Sindh and visited a tertiary care hospital for various complaints were selected at random. Their parents were asked to fill in a questionnaire regarding the symptoms and risk factors of NE. The data were gathered over a period of three months, starting from June 2019 to August 2019. The data were then analyzed to draw associations between the findings.

Results

Out of the total 1500 participants, 570 (38%) were male and the remaining 930 (62%) were female. Among these, the majority with NE were males (70%) and children between the age of six and seven years (65%). The first and fourth to fifth born were more likely to develop symptoms of NE. There was also a positive association between family history (P=0.003), delayed milestones (0.001), psychological problems (0.005), and urinary tract infection (P=0.001). However, a child suffering from chronic illness, parasitic infection, or anemia did not have a significant relationship with developing NE.

Conclusion

The total prevalence of NE among rural areas was 40%, which was higher than in urban areas. This could be due to limited awareness among parents and limited healthcare facilities to manage the condition in rural areas. However, it is important to identify the symptoms of NE earlier among children to reduce the impact it leaves on them.

## Introduction

Nocturnal enuresis (NE) is an involuntary urinary incontinence that occurs during sleep [1]. Voluntary micturition matures with the neurological development of a child. By the age of three to four years, the child learns to void voluntarily and by four years of age, it has gained urinary continence similar to adults [2]. Hence, NE is a condition where a child should be able to voluntarily control voiding but is unable to do so. Studies have shown that NE is a multifactorial condition, with genetic, developmental, and psychological factors contributing to its pathophysiology [3]. Among some of the well-known causes are electrolyte imbalance, decreased antidiuretic hormone (ADH) level, and reduced bladder capacity [4-5]. According to the International Children's Continence Society (ICCS), it is observed in children of more than five years of age but remains underdiagnosed until the age of seven to eight years [1].

NE is classified into primary NE (PNE) and secondary NE. In PNE, the child never gains complete urinary control and wets his or her bed with gaps of less than six months, whereas in secondary NE, the child remains dry for more than six months at length [1]. PNE is the most common type and is thought to be caused by a genetic predisposition towards the condition or developmental delays in neurological maturation [6]. Primary nocturnal enuresis is then classified into monosymptomatic and non-monosymptomatic NE depending on the associated symptoms such as dysuria, suprapubic pain, and daytime incontinence that are only present in non-monosymptomatic NE [1]. As the child grows older, these symptoms appear to resolve by themselves at about 15%-20% per year and hence reduce to a prevalence of 1%-2% by the age of 18 years as seen in adults [7]. On the contrary, enuretic children are two to six times more likely to develop psychiatric issues due to the constant low self-esteem, embarrassment, and aggression buildup, which can also lead to poor performance in school life [8].

Consequently, it is important to assess the prevalence of NE so that the children dealing with this condition can be given proper attention and management instead of being neglected. Though this condition does not have any long-term functional abnormalities related to it, it has shown to cause psychological disturbances in the child and their families as discussed above.

Previous prevalence studies on NE vary in different provinces of Pakistan and range from as high as 43% within an urban city, Karachi, to around 25% in the rural areas of Sialkot [9-10]. The objective of our study is to evaluate the prevalence, risk factors, and provided management to children dwelling in rural areas of Sindh province, Pakistan.

## Materials and methods

A cross-sectional study was conducted among children visiting the outpatient department (OPD) of Dr. Ruth K. M. Pfau Civil Hospital in Karachi from June to August 2019. It is a tertiary care hospital run by government authorities to provide free healthcare facilities to people belonging to low socioeconomic backgrounds or the rural parts of the Sindh province. All three to 12-year-old children belonging to the rural parts of Sindh visiting the OPD for any reason were included in the study. The purpose of our study was to determine the prevalence of NE, its risk factors, and the management provided to children with these complaints.

A questionnaire was built to carry out the survey. It included questions related to biodata (age, gender, birth order of child and address), symptoms of enuresis (frequency of NE, sleeping pattern, daytime symptoms), risk factors (family history of the condition, delayed development, comorbidities), and any management steps taken for the condition. All questions related to NE were drafted using available guidelines [1]. It was then tested on a sample of 50 children who met the criteria set for the study. The data from these inputs were analyzed and 45% of children among this pilot sample were found to have symptoms of NE. Using these figures, a sample size of 1497 was calculated at a confidence interval of 99.99%. However, for statistical convenience, a total of 1500 participants were recruited. The questionnaire was reviewed and modified accordingly.

The study protocol was approved by the ethical review board of the Dow University of Health Sciences. The participants were selected at random by convenient sampling. Each questionnaire included a consent form and was signed by the parents of the child. All participants were asked to fill the forms through self-administration or the forms were filled by the researcher through small interview sessions. The forms were translated into the native language (Urdu or Sindhi) for convenience. The data collected were analyzed using the Statistical Package for the Social Sciences (SPSS) version 19 and were formatted into tables. The qualitative data were arranged into frequencies and percentages. Multiple tests were applied to form associations within the results.

## Results

Out of the total 1500 participants, 570 (38%) were male and the remaining 930 (62%) were female as illustrated by Table 1 which depicts the demographic features of study participants. Among these, the majority, that is, 675 children (45%) were between the ages of six and seven years, followed by 450 children (30%) between the ages of three and five years, 255 (17%) were 11 years and above, and 120 (8%) were between eight and 10 years of age. Eight-hundred twenty-five (55%) parents knew about nocturnal enuresis, and of these, 600 (40%) knew about its causes and had children suffering from it. The majority (180; 12%) believed that the cause of NE is a weakness in the muscles of the lower urinary tract, and others (150; 10%) said that the damage to the urinary tract or nerves that control the urinary system is responsible for this condition. Moreover, 105 (7%) parents believed that underlying psychological problems could lead to NE, while the rest of them believed that urinary tract infection (45; 3%), hereditary reason (45; 3%), or anemia (30; 2%) could be causing these symptoms.

**Table 1:** Depicts demographic features, parents/caregivers’ knowledge of nocturnal enuresis and the prevalence of nocturnal enuresis among the studied participants

	Frequency N=1500	Percent
Child's age ( in years)		
3-5	450	30.0
6-7	675	45.0
8-10	120	8.0
≥11	255	17.0
Child's sex		
Male	570	38.0
Female	930	62.0
Birth order of the child		
1	495	33.0
2	345	23.0
3	195	13.0
4-5	240	16.0
≥6	225	15.0
Participants know about nocturnal enuresis		
Yes	825	55.0
No	675	45.0
Participants know about it causes		
Yes	600	40.0
No	900	60.0
Identified causes of nocturnal enuresis (by participants)		
Weakness in the muscles of the lower urinary tract	180	12.0
Problems or damage of the urinary tract or nerves that control the urinary system	150	10.0
Psychological problems	105	7.0
Urinary tract infections	45	3.0
Hereditary	45	3.0
Anemia	30	2.0
Irritability	30	2.0
Pregnancy and birth-related causes	15	1.0
Having a child suffering from nocturnal enuresis		
Yes	600	40.0
No	900	60.0

From the 600 children who had NE, as indicated by Table 2, which highlights their features, a total of 360 (60%) of them had both day and night incontinence while 240 (40%) had only complained of wetting the bed at night. The frequency of these symptoms ranged between five and seven times a week (390; 65%), three to four times a week (120; 20%) to one to two times a week (90; 15%). To combat the situation, 88% of the mothers were keen to wake the child up at night to urinate and 48% also reported that the child had improvement in his or her symptoms if the fluid intake before bedtime was restricted. However, due to the persistent nature of the condition, 510 (85%) children out of 600 felt embarrassed about having this problem. Many parents had sought treatment of nocturnal enuresis, and 40% of them stated that they were able to achieve improvement in the symptoms of their child's condition. Some of the popular methods adopted were behavioral modifications (378; 63.%), pharmacological treatments (108; 18%), bed-wetting alarms (60; 10%), and exercises to strengthen the bladder muscles (48; 8%).

**Table 2:** Illustrates nocturnal enuresis-related features among the studied participants

Variables	Frequency N=600	Percentages
Time of enuresis		
At night only	240	40.0
Day and night	360	60.0
Improvement of decreasing fluids intake before sleeping	288	48.0
Frequency per week		
1-2	90	15.0
3-4	120	20.0
5-7	390	65.0
Mother keen to wake the child at night to urinate	528	88.0
The problem causes embarrassment and social shame to the child	510	85.0
Seeking medical advice	390	65.0
Type of provided treatment		
Pharmacological treatment	108	18.0
Surgery	6	1.0
Exercises to strengthen the bladder muscles	48	8.0
Bedwetting alarm	60	10.0
Behavioral modification	378	63.0
Improvement of nocturnal enuresis on different types of treatment	240	40.0

Furthermore, the results showed a positive association of NE with the age of the child (P=0.04), with the highest prevalence in children between six and seven years of age as demonstrated in Table 3. Furthermore, gender (P=0.01), gestational age (P=0.02), type of delivery, that is, C-section or vaginal delivery, and hospital admission after delivery (P=0.001) were all found to have a positive association. The first and fourth to fifth born (P=0.007) are more likely to develop symptoms of nocturnal enuresis with a prevalence of 27% and 25%, respectively. A positive family history (P=0.003) within the parents or siblings suffering from the same condition also leads to more chances of the child developing NE. Some causes like urinary tract infections (UTIs), diabetes type 1, psychological problems, and delayed milestones were all found to be positively associated. However, a child suffering from chronic illness, parasitic infection, or anemia did not have a significant relation to developing NE.

**Table 3:** Depicts the risk factors of nocturnal enuresis among the studied participants

Variables	Responses	Nocturnal	Enuresis	Total	P-value
		Yes (N=600)	No (N=900)	(N=1500)	
Child age in years	3-5	20.0% (120)	36.6% (330)	30.0% (450)	0.04*
	6-7	65.0% (390)	31.6% (285)	45.0% (675)	
	8-10	8.5% (51)	7.6% (69)	8.0% (120)	
	≥11	6.5% (39)	24.0% (216)	17.0% (255)	
Sex	Male	70.0%(420)	16.7%(150)	38.0% (570)	0.01*
	Female	30.0%(180)	83.3%(720)	52.0% (930)	
Gestational age (in months)	9	85.0% (510)	93.3% (840)	90.0% (1350)	0.02*
	8	6.0% (36)	4.3% (39)	5.0% (75)	
	7	8.0% (48)	1.3% (12)	4.0% (60)	
	≤6	1.0% (6)	0.6%(9)	1.0% (15)	
Type of delivery	Vaginal	70.0% (420)	85.0% (765)	79.0% (1185)	0.001*
	Cesarean section	30.0% (180)	15.0% (135)	21.0% (315)	
Hospital admission after delivery	No	89.0% (534)	94.0% (846)	88.0% (1380)	0.001*
	Yes	11.0% (66)	12.7% (114)	12.0% (180)	
Sibling suffering from the same condition	No	68.0% (408)	83.0% (747)	77.0% (1155)	0.003*
	Yes	32.0% (192)	17.0% (153)	23.0% (345)	
Birth order of the child	1	27.0% (162)	37.0% (333)	33.0% (495)	0.007*
	2	16.0% (96)	27.7% (249)	23.0% (345)	
	3	18.0% (108)	9.7% (87)	13.0% (195)	
	4-5	25.0% (150)	10.0% (90)	16.0% (240)	
	>5	14.0% ( 84)	15.7% (141)	15.0% (225)	
History of parents with same condition during their childhood	No	79.0% (474)	87.3% (786)	84.0% (1260)	0.003*
	Yes	21.0% (126)	12.7% (114)	16.0% (240)	
The child has chronic illness	No	88.0% (528)	91.3% (822)	90.0% (1350)	0.572
	Yes	12.0% (72)	8.7% (78)	10.0% (150)	
Anemia	No	88.0% (528)	93.0% (837)	91.0% (1365)	0.234
	Yes	12.0% (72)	7.0% (63)	9.0% (135)	
Parasitic infestation	No	89.0% (534)	94.0% (846)	92.0% (1380)	0.767
	Yes	11.0% (66)	6.0% (54)	8.0% (120)	
Diabetes type I	No	93.0% (558)	99.7% (897)	97.0% (1455)	0.005*
	Yes	7.0% (42)	0.3% (3)	3.0% (45)	
Urinary tract infection	No	89.0% (534)	99.0% (891)	95.0% (1425)	0.001*
	Yes	11.0% (66)	1.0% (9)	5.0% (75)	
Psychological problems	No	79.0% (474)	97.3% (876)	90.0% (1350)	0.005*
	Yes	21.0% (126)	2.7% (24)	10.0% (150)	
Delayed milestones	No	92.0% (552)	97.0% (873)	95.0% (1425)	0.001*
	Yes	8.0% (48)	3.0% (27)	5.0% (75)	

## Discussion

Nocturnal enuresis is one of the conditions whose prevalence varies throughout the world, being 10.2% in Iran, 16.2% in Turkey, 18.9% in Australia, and 23.2% in Nigeria [11-14]. The prevalence of NE in our study was found to be 40% in children aged between three and 12 years living in the rural areas of the Sindh province, which is higher as compared to these. However, studies conducted in Saudi Arabia and Jamaica reported a higher prevalence of 63.6% and 50%, respectively [15-16]. This higher prevalence noted could be because of our study setting, as it is observed that the prevalence of NE is higher in rural than urban areas. It could also be due to the reason that people living in rural areas generally belong to a lower socioeconomic class and NE is generally higher among this group [17]. Moreover, low parental education levels or unfavorable social conditions leading to psychological stress in children that predisposes them to develop NE could also be some of the reasons why the prevalence is higher in rural areas [18]. In our study, the prevalence of NE in boys (70%) was more than double that in girls (30%). This finding is consistent with earlier studies that found the prevalence in boys to be 1.65 times that in girls [19]. Another study stated that the ratio is as high as 5:1 of boys to girls with NE [20]. Since voluntary urinary continence develops as the child matures and as boys mature slower than girls do; hence, this can explain the higher prevalence in them [21]. However, some studies showed no difference among both genders [15]. The symptoms of NE regress as the child gets older, and its prevalence usually decreases with age. Our study supported this, as the prevalence peaked at 65% in children between six and seven years of age and decreased in older age groups. This finding is similar to prior studies, which also reported the highest prevalence among six to seven-year-old participants [15,22].

We also found a significant association between birth order and the child developing symptoms of NE. The first and fourth to fifth born were more likely to have this condition. There is some evidence that in large families, the younger ones have lower self-esteem as compared to their older siblings [23]. This may be the reason why those born later in large families are enuretic, as low self-esteem is known to be a risk factor for this condition [24]. Increased family size is also linked to children being more frequently enuretic at night [23]. Furthermore, having a positive family history (in parents or siblings) was also a significant association in our study.

According to our results, 60% of enuretic children had daytime and nighttime symptoms, which is congruent with a Saudi study where 55% of children had such symptoms [15]. However, one study reported a lower prevalence of 18% of daytime symptoms [25].

In this study, 65% of participants wet their beds five to seven times per week. This finding is higher than the one found earlier in the urban side of Karachi where 30% of children wet their bed every night and 30% more than three nights a week [22]. This could be because the participants in our study are from a rural setting where treatment facilities are limited and awareness is minimal due to low parental education. Other studies reported a lower frequency, with 17% children wetting their bed at least twice a week and 8.2% wetting every night, and in China, 24.60% of children wet their bed every night [26-27].

The majority of parents in this survey adopted a behavioral modification to manage the condition of their child by waking them up at night for urinating and restricting their fluid intake before sleeping. Interestingly, a study reported that half of the families were able to manage their child’s symptoms using these modifications and hence using such methods can help the child overcome their problem [27]. Other methods adopted by parents in our study included pharmacological therapy, bedwetting alarms, and exercises to strengthen the urinary bladder.

## Conclusions

Our study found a high prevalence of nocturnal enuresis in children aged between three and 12 years belonging to rural areas of Sindh. Boys were more likely to present with symptoms of NE, and six to seven years old made up most of the enuretic population in our study. A positive family history, birth order, and children with a psychological or hereditary link were all positively associated with NE. Hence, these risk factors should be evaluated earlier in children and managed accordingly to overcome any psychological or health impact on the development of the child.
